# Supplementary material for: The Fate of Cooperation during Range Expansions
Source: PLoS Comput Biol. 2013 Mar 28;9(3):e1002994. doi: 10.1371/journal.pcbi.1002994 (PMC3610630; doi:10.1371/journal.pcbi.1002994)
Supplement: Text S1 — contains the derivation of the boundary conditions for equation (15), the derivation of equation (18), and the discussion of the effects of effective advection and growth on the ability of defectors to travel faster during a range expansion with cooperators than during an invasion into a population of cooperators. (PDF) [file pcbi.1002994.s001.pdf]

Supporting Text
The fate of cooperation during range expansions

Kirill S. Korolev[1,*]
1 Massachusetts Institute of Technology, Department of Physics, Cambridge, MA 02139
∗ E-mail: papers.korolev@gmail.com

In this Supporting Text, we first expand our discussion of the boundary conditions for equation (16). We then outline the derivation of the exact solution, given by equation (18), for the transition from non-splitting to splitting behavior in a special case of equation (12). Finally, we show that both effective advection and effective growth can allow defectors to expand faster in mixed waves than they can do by invading cooperators.

Boundary conditions for equation (16)

Here, we show that $\psi(\pm\infty) = 0$ are the proper boundary conditions for equation (16). Since the boundary conditions for $f(\zeta)$ are $f(-\infty) = 1$ and $f(+\infty) = 0$, it is sufficient to show that $u(-\infty) > 0$ and $u(+\infty) \leq 0$; see equation (15). The former condition follows from the fact that $v_{\mathrm{c}} > 0$ and $v_{\mathrm{a}}(-\infty) = 0$. The latter condition follows from the fact that $v_{\mathrm{a}}(+\infty) \geq v_{\mathrm{c}}$, which we prove below.

The density profile at large ζ decays exponentially as $e^{-\gamma\zeta}$; moreover, $\gamma \geq \sqrt{G_c(0,0)/D}$ for a weak Allee effect [1]. We then substitute this scaling form in the equation (9) for $c(t,x)$, change to the comoving reference frame, and require that the wave profile does not depend on τ. This calculation yields

$$v_{\mathrm{c}} = D\gamma + \frac{G_c(0,0)}{\gamma}. \tag{1}$$

This expression for v_{c} can now be compared to the advection velocity $v_{\mathrm{a}} = -2D\partial \ln[c(t,x)]/\partial x = 2D\gamma$. For a strong Allee effect, $G_c(0,0)$ is negative and, therefore, $v_{\mathrm{a}} > v_{\mathrm{c}}$. For a weak Allee effect, $v_{\mathrm{a}} - v_{\mathrm{c}} = D\gamma - \frac{G_c(0,0)}{\gamma} \geq 0$, where the last inequality follows from $\gamma \geq \sqrt{G_c(0,0)/D}$; see [1].

Derivation of equation (18)

Here, we derive equation (18). Since, for $G_f(c,f)$ decreasing with f, wave splitting is affected only by the linearization of $G_f(c,f)f$ for small f, we are free to replace $G_f(c,f)f$ with any other function that has identical behavior for $f \to 0$. It is particularly useful to approximate $G_f(c,f)f$ with a piece-wise linear function because this allows for an exact solution for $f(t,x)$ everywhere in space, which captures the qualitative dependence of the solution on the parameters of the model. In contrast, eigenfunctions of $\mathcal{L}$ only describe the behavior of $f(t,x)$ close to the front and only up to a normalization factor. To this purpose, we redefine $G(c,f)f$ as

$$G_f(c,f)f = g_f(c)f^*(c)\min\{f, f^*(c) - f\}. \tag{2}$$

Note that this redefinition does not have a fixed point at $f = 1$, which is appropriate as long as we are not interested in cooperators invading defectors.

We now turn to equation (12), which can be further simplified by the change of variables from ζ to $\varphi = c/K$ using equation (4). The result reads

$$\varphi^2(1-\varphi)^2 f'' + 2\varphi(1-\varphi)\left(1 - 2\varphi + \frac{c^*}{K}\right) + \frac{2}{g_c K^2}[G_f(K\varphi, f) - \lambda]f = 0, \tag{3}$$

where primes now denote derivatives with respect to φ. We further assume that $c^* = 0$ and obtain that

$$[\varphi^2(1-\varphi)^2 f']' = -\frac{2}{g_c K^2} G_f(K\varphi, f)f, \tag{4}$$

where we also set $\lambda = 0$ because λ vanishes at the splitting transition. We now use equation (S2) and the assumption that $f^*(\varphi) = f^*$ for $\varphi > \bar{\varphi} \equiv \bar{c}/K$ and $f(\varphi) = 0$ for $\varphi < \bar{\varphi}$, i.e. defectors are not viable at very low densities. The right hand side of equation (S4) is then proportional to f for $f < f^*/2$ and to $f^* - f$ for $f > f^*/2$; therefore, further simplifications occur upon integrating both sides of equation (S4) over φ and defining a new variable $F = \int f d\varphi$ for $f < f^*/2$ and $E = \int (f^* - f) d\varphi$ for $f > f^*/2$. For example, for the region where $f < f^*/2$ and $\varphi > \bar{\varphi}$, we obtain

$$\varphi^2(1-\varphi)^2 F'' = -\frac{2g_f f^*}{g_c K^2} F \tag{5}$$

The general solution of this equation is given by

$$\begin{aligned} F &= C_1 \varphi^{\alpha}(1-\varphi)^{1-\alpha} + C_2 \varphi^{1-\alpha}(1-\varphi)^{\alpha}, \;\; \text{with} \\ \alpha &= \frac{1}{2}\left(1 + \sqrt{1 - \frac{8 g_f f^*}{g_c K^2}}\right). \end{aligned} \tag{6}$$

Similarly, for $f > f^*/2$, we find

$$\begin{aligned} E &= C_3 \varphi^{\beta}(1-\varphi)^{1-\beta} + C_4 \varphi^{1-\beta}(1-\varphi)^{\beta}, \;\; \text{with} \\ \beta &= \frac{1}{2}\left(1 + \sqrt{1 + \frac{8 g_f f^*}{g_c K^2}}\right). \end{aligned} \tag{7}$$

Then, the solution for $f(\varphi)$ is given by E' from equation (S7) for $f > f^*/2$ and by F' from equation (S6) for $f < f^*/2$ and $\varphi > \bar{\varphi}$. The four constants C_1, C_2, C_3, and C_4 and the matching point $\varphi_{1/2}$ are determined by the boundary conditions $f(-\infty) = f^*$ and $f(\bar{c}/K) = 0$ and the matching conditions at $\varphi_{1/2}$, where $f(\varphi_{1/2} - 0) = f(\varphi_{1/2} + 0) = 1/2$ and $f'(\varphi_{1/2} - 0) = f'(\varphi_{1/2} + 0)$. Upon using four of the five conditions, one can easily express the four constants in terms of $\varphi_{1/2}$ because the conditions are linear with respect to C_1, C_2, C_3, and C_4. The remaining condition determines $\varphi_{1/2}$ and can also be solved exactly:

$$\begin{aligned} \varphi_{1/2} &= \frac{\gamma \bar{\varphi}}{1 - (1-\gamma)\bar{\varphi}}, \;\; \text{where} \\ \gamma &= \left[\frac{(\beta - \alpha)(\alpha - \bar{\varphi})}{(\beta + \alpha - 1)(1 - \alpha - \bar{\varphi})}\right]^{\frac{1}{2\alpha - 1}}. \end{aligned} \tag{8}$$

We then obtain the condition for splitting, stated in equation (18), by requiring that γ is real, and $\varphi_{1/2} > \bar{\varphi}$. Note that, $\varphi_{1/2} \to 1$ as the splitting state is approached, say, by increasing $\bar{c}$. Since $\varphi \to 1$ corresponds to $\zeta \to -\infty$, we see that the wave of defectors lags more and more behind the wave of cooperators. As we show in the main text, this is a general result, and the front is populated mostly by the cooperators close to the splitting transition. As a consequence, the expansion velocity at the splitting transition is given by equation (3).

Increased spreading through effective advection and growth

Here, we demonstrate the effects of effective advection and growth on the ability of defectors to keep up with cooperators. When ecological dynamics are not affected by the evolutionary dynamics, the ability of defectors to follow cooperators in a mixed wave is mathematically equivalent to the ability of a Fisher

wave to follow a moving boundary between good and bad conditions. In particular, the assumption that $f = 0$ for $c < \bar{c}$ makes this moving boundary absorbing. One can easily show that classic Fisher waves can only follow an absorbing boundary that moves with a velocity smaller than the Fisher velocity. Here, we show that even boundaries moving with higher velocities can be followed provided there is an effective advection or growth. This analysis illustrates the role of the term $2D\frac{\partial \ln(c)}{\partial x}\frac{\partial f}{\partial x}$ in equation (10) and suggests that both effective advection and growth could be useful management strategies to maintain genetic diversity during range expansions or to help species shift in response to a rapid climate change.

To avoid confusion with the earlier discussion, we introduce new notation. Let v be the velocity of the absorbing barrier, and g is the per capita growth rate. The Fisher velocity is then $2\sqrt{gD}$, where D is the effective diffusion constant. When $v > 2\sqrt{gD}$, the population cannot keep up with the barrier without external interventions. One possible intervention is to increase g by a factor $1+\delta$, say by supplying extra food, in a region of length a behind the barrier, which is analogous to the effective growth discussed earlier. Another possibility, analogous to effective advection, is to move the population with velocity u in a region of length a behind the front, say by capturing and transporting individuals within the population. The analysis of both of these scenarios follows the same procedure as the derivation of (18), but is much simpler because the resulting linearized equations have constant coefficients; therefore they can be easily solved by the standard methods. Below we just summarize the results.

For effective growth, we find that barriers moving with any velocity can be followed, but the region of effective growth should be sufficiently large:

$$a > \frac{2D}{\sqrt{4gD(1+\delta)-v^2}}\left[\pi - \arctan\left(\frac{\sqrt{4gD(1+\delta)-v^2}}{\sqrt{v^2-4gD}}\right)\right]. \tag{9}$$

Note that the minimal size is finite when $v = 2\sqrt{gD}$, and that it decreases only as $\delta^{-1/2}$ for large δ. This suggests that there is an optimal size a that minimizes δa, which could be interpreted as the total cost of the intervention.

For effective advection, we also find that barriers moving with any velocity can be followed provided $2\sqrt{gD} + u > v$ and the region of advection is sufficiently large:

$$a > \frac{2D}{\sqrt{4gD-(v-u)^2}}\left[\pi - \arctan\left(\frac{\sqrt{4gD-(v-u)^2}}{u+\sqrt{v^2-4gD}}\right)\right]. \tag{10}$$

Similar to the previous case, minimal a is finite when $v = 2\sqrt{gD}$. More importantly, the minimal size is the smallest when u and v are about equal. Indeed, when $v \gg u$, advection has a small effect on the dynamics, while, when $u \gg v$, the point where the region of advection ends becomes an effectively absorbing boundary because organisms entering advection region are quickly moved towards the front of the wave.

References

1. Van Saarloos W (2003) Front propagation into unstable states. Phys Rep 386: 29–222.
